# Association of circulating microRNA-122 with presence and severity of atherosclerotic lesions

**DOI:** 10.7717/peerj.5218

**Published:** 2018-07-04

**Authors:** Yu-Long Wang, Wen Yu

**Affiliations:** 1 Department of Cardiology, Haiyan People’s Hospital, Haiyan, Zhejiang, China; 2 Department of Geriatric, Zhejiang Provincial People’s Hospital, People’s Hospital of Hangzhou Medical College, Hangzhou, Zhejiang, China

**Keywords:** microRNA-122, Coronary heart disease, Biomarker, Atherosclerosis

## Abstract

**Objective:**

MicroRNA (miR)-122 is highly expressed in the liver, where it has been implicated as a regulator of fatty-acid metabolism. A recent study reported that miR-122 plays a role in pathogenesis of atherosclerosis; however, whether it connects with severity of atherosclerotic lesion is still controversial. We therefore investigated the association between miR-122 expression and presence and severity of coronary atherosclerotic plaque.

**Methods:**

During January–November 2017, we included 300 patients with coronary heart disease (CHD) and 100 subjects as the control group. MiR-122 content was detected by quantitative real-time polymerase chain reaction. MiR-122 level was identified in all subjects, and the Spearman correlation between miR-122 and severity of atherosclerosis was analyzed.

**Results:**

Patients with CHD had higher miR-122 expression than in control group (2.61, 0.91–8.86 vs. 1.62, 0.71–3.45, *p* < 0.001). Gensini score was significantly associated with miR-122 expression (*r* = 0.7964, *p* < 0.001). The odds ratio of miR-122 solely was 0.12 (95% CI [0.05–0.43]) and factors such as cholesterol, triglyceride together with miR-122 level were closely associated with atherosclerosis (all *p* < 0.001).

**Conclusions:**

The serum level of miR-122 may be used to differentiate between mild and severe coronary atherosclerotic lesion. Use of this marker might allow non-invasive diagnosis the degree of coronary atherosclerosis.

## Introduction

MicroRNA (miRNAs) is a well-known marker of atherosclerosis and a good predictor for future risk of cardiovascular events. Previous studies indicated that abnormal expression levels of plasma miRNAs is helpful for improving the discrimination of coronary heart disease (CHD) (Corsten et al., 2010; Li et al., 2017; Zhang et al., 2017). There is clear evidence that an independent relationship exists between plasma cholesterol levels and the risk for atherosclerotic lesion (Jensen et al., 2017). The concept that several miRNAs are potential indicators for lipid metabolism has expanded very quickly in recent decade (Cheng et al., 2017; Gadde & Rayner, 2016).

MiR-122 is the most abundant liver-specific miRNA (250,000 copies/hepatocyte) that is evolutionarily conserved accounting for up to 70% of total adult liver miRNAs (Pirola et al., 2013). Under normal conditions, miR-122 is released into the circulation in a constant manner via hepatic exosomes, a mechanism that is modulated by statins (Huang et al., 2013). MiR-122 inhibition by antisense oligonucleotide reduced plasma levels of cholesterol and triglyceride, and it plays a role in cholesterol and free fatty-acid (FFA) metabolism, and the role of circulating miR-122 in predicting human nonalcoholic fatty liver disease has been replicated in several studies (Chai et al., 2017; Esau et al., 2006). Willeit et al. (2016) reported that miR-122 had a known effect on lipid metabolism, making it a promising candidate biomarker of lipid homeostasis.

In humans, it has been suggested that miR-122 may have adverse metabolic effects and may be associated with metabolic diseases (Naveed et al., 2017). A previous study also reported that liver-specific miR-122 level is significantly higher in plasma of patients with CHD (Li et al., 2017). However, evidence from existing epidemiological studies is sparse and has important limitations. Importantly, previous studies had limited sample sizes and hence were unable to inform about association of circulating miR-122 with the development of new-onset coronary atherosclerosis outcomes over time (Aryal et al., 2017).

In the present study, we investigated whether miR-122 level could be utilized in differentiating atherosclerotic lesion in certain population. In addition, we investigated the relationship between severity of atherosclerosis and expression level of miR-122.

## Methods

### Study population

A total of 400 people, who underwent invasive coronary angiography (ICA) for suspected CHD were included at the Zhejiang Provincial People’s Hospital during January–November 2017. Altogether, we recruited 300 patients with CHD and 100 subjects as the controls (coronary vessel stenosis <50% or normal). The inclusion criteria of patients with CHD included: (I) older than 18 years; (II) vessel stenosis ≥50% in at least one coronary artery confirmed by ICA; (III) individuals who had signed informed consents. We excluded the follow subjects: (I) heart failure at class IV; (II) anemia (hemoglobin <10 g/dL); (III) any chronic illness, such as cancer, liver or renal failure; (IV) patients who denied to participate in the study.

Baseline characteristics were collected at admission, including age, gender; and history of hypertension, diabetes mellitus and smoking. Laboratory parameters including total cholesterol (TC), low-density lipoprotein cholesterol (LDL-C), high-density lipoprotein cholesterol, triglyceride (TG), uric acid, white blood cell and creatinine were also collected.

The study design and methods complied with the Declaration of Helsinki and was approved by the Ethics Committee and Institutional Review Board of Zhejiang Provincial People’s Hospital (KY-20170003). Full written informed consent was obtained from all subjects.

### Atherosclerosis assessment

Invasive coronary angiography (GE Digital Radiography System) was conducted in all study population. Coronary artery was cannulated by means of radial approach with 6F catheters. All lesions were evaluated in multiple projections as previously described (Chan et al., 2017; Samman et al., 2018). Degree of coronary atherosclerosis was evaluated by two cardiologists according to the luminal narrow in arteries. The severity of the coronary stenosis was in accordance with the Gensini score (Morisawa et al., 2017; Wang et al., 2018).

### Quantitative assessment of miR-122

All study individuals were asked to provide a blood sample and complete a risk factor questionnaire at the initiation of the study. Blood samples (5 ml) were collected from the antecubital vein on the following morning after admission. All samples were then transferred to EDTA and citrate (3.2 and 3.8%) blood collection tubes. The protocol of processing the blood samples was used to as previously described (Zhang et al., 2017): clotting at room temperature for 30 min to 2 h, then centrifugation at 1,200 × *g* for 10 min at 4 °C to remove debris and other large cells. The supernatant was then isolated and centrifuged at 16,000 × *g* for 15 min at 4 °C to obtain the serum. All serum were stored at −80 °C until further analysis was conducted.

Total RNA was extracted from 0.3 mL of serum by TRIzol LS reagent (Invitrogen, Carlsbad, CA, USA). The expression of miR-122 was evaluated by quantitative real-time polymerase chain reaction (qRT-PCR) analysis. PCR was conducted on cDNA generated from 50 ng of total RNA using the TaqMan^®^ MicroRNA Assay kit (Applied Biosystems, Foster City, CA, USA) in accordance with the manufacturer’s instructions. Then qRT-PCR was performed in triplicate using an ABI Prism 7500 sequence detection system (Applied Biosystems, Foster City, CA, USA). RNU6B was performed as a miRNA internal control. The amplification reactions were incubated at 95 °C for 30 min followed by 40 cycles at 94 °C for 15 s, 55 °C for 30 s, and 70 °C for 30 s.

RNU6B was used as endogenous control. The expression level of the miRNA-122 was quantified in accordance with the cycle threshold (Ct) method. Ct was regarded as the number of cycles needed for the fluorescent signal in crossing detection threshold. Relative gene expression was calculated by comparing the cycle times for target gene. The relative expression level between miR-122 and endogenous control was calculated as follows: relative miR-122 expression = 2^−(ΔCt sample − ΔCt RNU6B)^.

### Statistical analysis

A Kolmogorov–Smirnov normality test was performed to examine whether the data showed normal distribution or not. The result indicated that all quantitative data did not comply with the normal distribution. Skewed data were expressed as median and range, and comparisons were performed using the Mann–Whitney *U* test. Categorical variables of gender, medical history and previous medication were compared between two groups using a Pearson chi-squared analysis. Spearman’s rho was used to determine the relationship between Gensini scores with miR-122 levels. A multivariable backward stepwise logistic regression approach was used to examine the relationship between traditional risk factors and coronary atherosclerosis. The odds ratio of miR-122 were tested in unconditional logistic regression.

All differences were considered significant at *p* < 0.05. Data were subjected to statistical analysis using SPSS 22.0 (SPSS Software Inc., Chicago, IL, USA) and GraphPad Prism 6.02 software (GraphPad Software Inc., La Jolla, CA, USA).

## Results

A total of 400 study subjects were included in accordance with the eligibility criteria. The present study included 300 subjects with CHD and 100 without CHD as the controls (ICA exclusion of CHD). Two groups were basically well-matched on separate index in baseline characteristics (Table 1). However, in CHD group, TG, TC as well as LDL-C were significantly higher (all *p* < 0.01).

**Table 1:** Baseline characteristics in patients with CHD and control group.

Characteristics	CHD (*n* = 300)	Control (*n* = 100)	*p* value
Age, median (range), y	59 (40–86)	61 (31–87)	0.531
Men, No. (%)	205 (68.3)	72 (72.0)	0.491
Medical history, No. (%)			
Hypertension	155 (51.6)	56 (56.0)	0.450
Diabetes	75 (25.0)	19 (19.0)	0.221
Current smoker	140 (46.7)	41 (41.0)	0.325
Previous medication, No. (%)			
β-blocker	26 (8.7)	11 (11.0)	0.484
ACEI/ARB	126 (42.0)	44 (44.0)	0.729
Statin	100 (33.3)	40 (40.07)	0.225
Biochemistry detection			
WBC count, × 10^9^/L, median (range)	6.52 (3.46–17.62)	6.22 (2.98–13.96)	0.212
HDL-C, mmol/L, median (range)	1.03 (0.57–1.88)	0.95 (0.47–3.73)	0.039
LDL-C, mmol/L, median (range)	1.93 (0.84–4.32)	2.41 (0.81–7.30)	<0.001
TG, mmol/L, median (range)	1.11 (0.42–2.78)	1.83 (0.38–6.27)	<0.001
TC, mmol/L, median (range)	3.26 (1.95–6.03)	3.84 (1.93–8.93)	<0.001
CR, μmol/L, median (range)	94 (53–319)	96 (59–241)	0.934
UA, μmol/L, median (range)	259 (108–476)	267 (117–500)	0.952

**Note:**

TG, Triglyceride; TC, Total cholesterol; CR, Creatinine; UA, Uric Acid; BMI, body mass index; HDL-C, High-density lipoprotein cholesterol; LDL-C, Low-density lipoprotein cholesterol; miRNA, micro-RNA.

**Figure 1:**
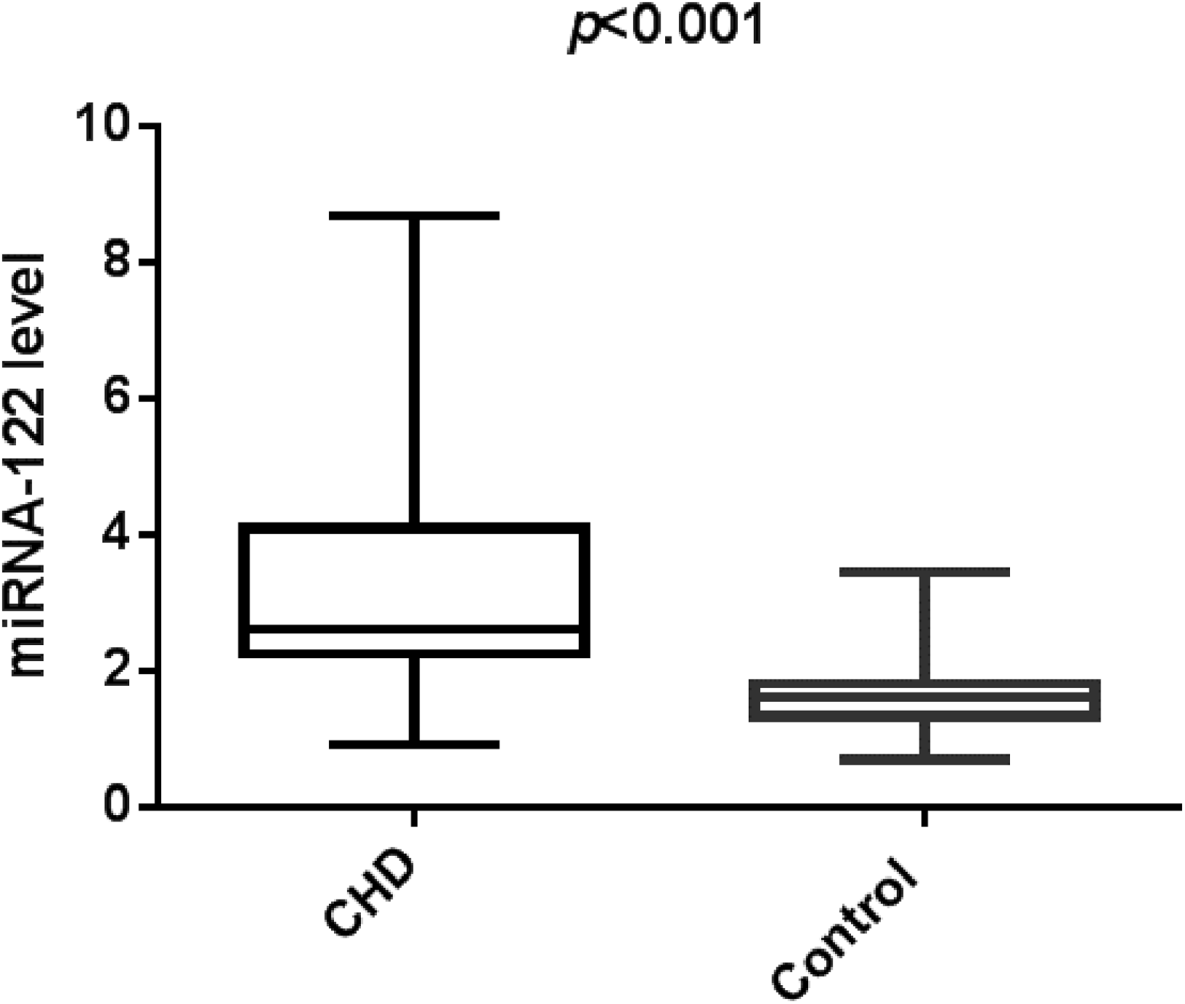
The relative expression of circulating miR-122 between the controls and CHD cases.

**Figure 2:**
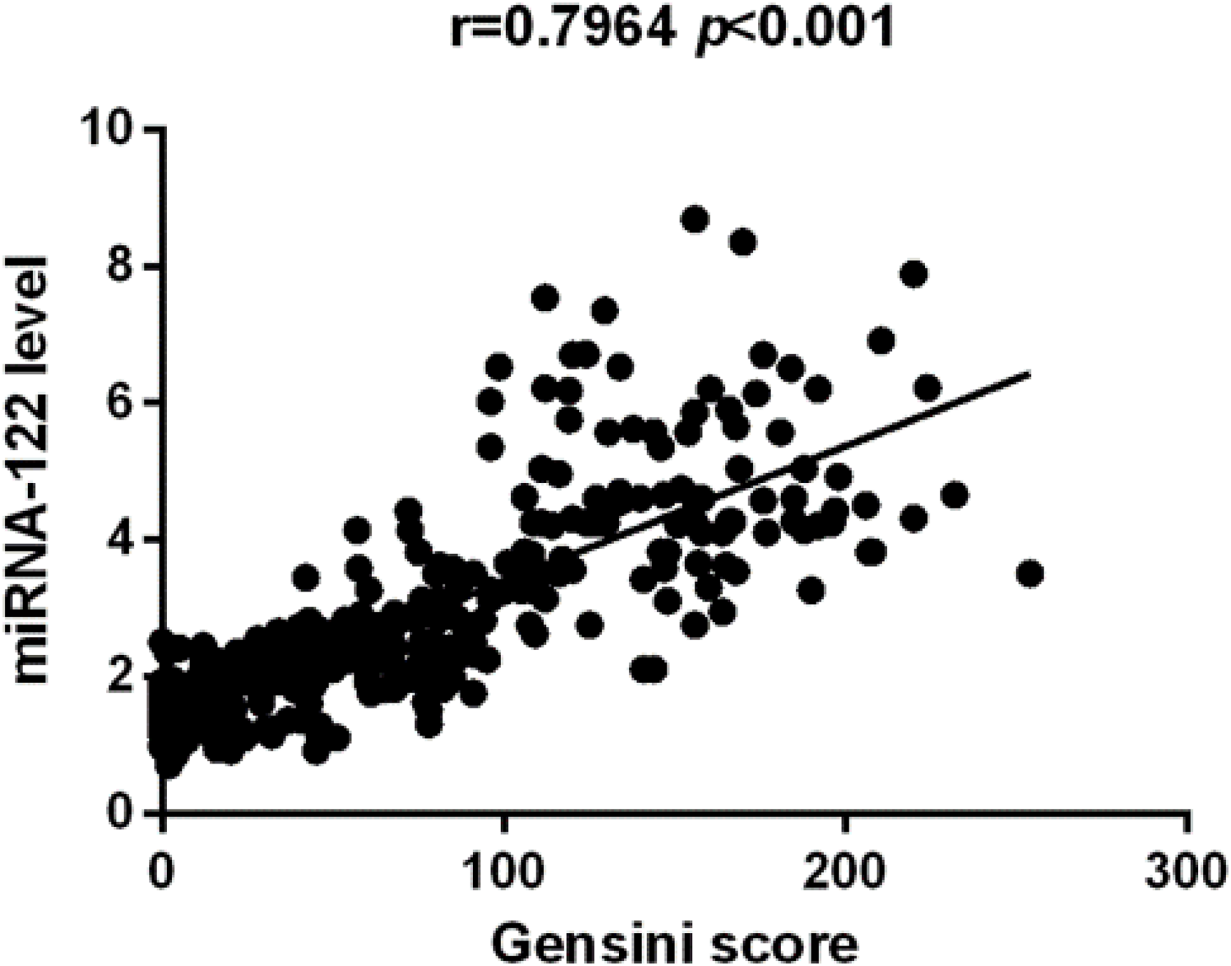
The Spearman correlation between relative miR-122 content and severity of atherosclerosis.

In this study, we identified that CHD group has increased serum miR-122. Figure 1 indicated that study population with CHD had significantly higher serum miR-122 level when compared with the control group (2.61 (0.91–8.68) vs. 1.62 (0.71–3.45), *p* < 0.001). Spearman’s rho correlation was applied due to non-normal distribution, there was a positive correlation between serum miR-122 and Gensini score (*r* = 0.7964, *p* < 0.001), indicating that miR-122 levels are correlated with the stage of coronary atherosclerotic lesions (Fig. 2). We further analyzed the correlations of plasma levels of miR-122 with TC and TG. As shown in Figs. S1 and S2, the plasma levels of miR-122 was positively correlated with TC, TG.

On basis of the result of multivariate regression analysis, six variables significantly associated with the presence of CHD (Table 2). The odds ratio of miR-122 solely was 0.12 (95% CI [0.05–0.43]), while after adjusting for the traditional risk factors, the odds ratios of miR-122 was 0.03 (95% CI [0.01–0.08]). The logistic regression analysis revealed that factors such as TC and TG together with miR-122 levels were closely associated with pathogenesis of the coronary atherosclerotic lesion (all *p* < 0.001).

**Table 2:** Multivariable model for predicting risk factor of coronary atherosclerosis.

	χ^2^ value	OR (95% CI)	*p* value
miRNA-122	42.92	0.03 (0.01–0.08)	<0.001
TC	6.46	0.31 (0.13–0.88)	0.011
TG	22.46	0.13 (0.06–0.31)	<0.001
LDL	6.50	0.27 (0.10–0.74)	0.011
HDL	26.20	3.47 (1.33–9.08)	<0.001
Creatinine	0.46	1.01 (0.98–1.03)	0.496
Age	1.33	1.03 (0.98–1.07)	0.783
Diabetes mellitus	0.08	0.86 (0.31–2.38)	0.557
Hypertension	0.03	0.93 (0.39–2.21)	0.868
Smoker	1.97	0.47 (0.16–1.35)	0.160
UA	0.05	0.99 (0.98–1.00)	0.825
Gender	4.33	0.31 (0.10–0.93)	0.037
WBC	0.83	1.10 (0.90–1.34)	0.363

**Note:**

TG, Triglyceride; TC, Total cholesterol; CR, Creatinine; UA, Uric Acid; WBC, White blood cell.

## Discussion

In the present study, we identified that miR-122 was significantly up-regulated in patients with atherosclerotic lesion, and the serum level of miR-122 positively correlated with atherosclerotic severity. Thus, we agree that miR-122 may regard as a robust biomarker for predicting atherosclerosis progression and perhaps is able to solve the dilemma of the usefulness of aminotransferases in deciding patients’ monitoring and ICA indication.

In recent years, emerging evidence has indicated that miRNAs play important roles in regulating lipid metabolism, and recent studies indicate that it may predict severity of atherosclerotic disease as well as cardiovascular event (Yu, Xu & Li, 2009; Zhang et al., 2017). In the healthy heart, miR-122 was expressed at very low levels or was undetectable, but increased in the border and ischemic areas of the AMI mice (D’Alessandra et al., 2010), in plasma of patients with acute heart failure (Corsten et al., 2010), and in plasma of the porcine cardiogenic shock model (Andersson et al., 2012). A previous study reported that human Agpat1 and Dgat1 were miR-122 target genes, implying that miR-122 involved in the TG synthesis pathway (Chai et al., 2017). Additionally, FFAs increased miR-122 expression in livers of mice by activating the retinoic acid-related orphan receptor alpha, and induced secretion of miR-122 from liver to blood (Chartoumpekis et al., 2012; Zhu & Fan, 2011). Taken together, these results emphasize the role of miR-122 in regulating lipid metabolism. Considering the indispensable role of hyperlipidemia in the onset and development of atherosclerotic lesions, dysregulation of lipometabolism-related miRNAs might also be related to the presence of coronary arteriosclerotic disease (CAD).

To date, the researches about specific connections between miRNA-122 level and severity of coronary atherosclerosis are scant. Our result are basically consistent with a study conducted by Gao et al. (2012), which indicated that plasma levels of miR-122 correlate with hyperlipidemia and CAD. However, in their study, only 155 CAD patients were recruited. Moreover, their result was only 0.265 in *r* value (*p* = 0.04) in defining obvious correlation between plasma miR-122 and Gensini score, which is with difficulty persuasive. It is possible that limited sample sizes and selection bias may account for the inconsistency between studies. Thus, a study with larger and different population is needed to elucidate the relationship between miR-122 and coronary atherosclerosis.

While some studies dispute its efficacy for diagnosing various types of liver disease, other research indicates that it may participate in the pathogenesis of metabolic syndrome. However, there is limited research into the use of miR-122 as an indicator for the presence and severity of atherosclerosis.

The coronary atherosclerotic severity was on basis of the Gensini score (Liu et al., 2017). As for the potential role of miR-122, we believe that the overall effect of miR-122 may further lead to the pathogenesis of atherosclerotic lesions, although the pathways involved in this regulation have not been fully elucidated.

In this study, to avoid the influence of relevant risk factor, we have recruited patients with similar conditions (such as medical history) in both groups at the beginning. Thus, baseline characteristics were not normal in our research; however, the baseline characteristics is not always perfectly in accordance with normal distribution in many researches, especially in laboratory data (Gräni et al., 2017; Núñez et al., 2018). In this case, the median best retains this position and is not as strongly influenced by the skewed values.

A major limitation was that this cross-section study does not guarantee the power to clarify a causal relationship between miR-122 content and presence of coronary atherosclerotic lesions.

## Conclusions

Taken together, the result expand our knowledge regarding the pivotal role of miR-122 in atherosclerosis. The serum level of miR-122 was increased in patients with CHD when compared with a healthy population, and this marker may also be used to differentiate between mild and severe coronary atherosclerotic lesion. These understandings could pave the way for the development of a novel biomarker for coronary atherosclerosis. Use of this marker may allow a certain indicator of atherosclerosis at a much lower cost than methods currently available. However, this was a single-center research; further study might be conducted to validate this non-invasive test for the diagnostic accuracy in CHD.

## Supplemental Information

10.7717/peerj.5218/supp-1 Supplemental Information 1 Figures S1 and S2.

10.7717/peerj.5218/supp-2 Supplemental Information 2 Raw data.

## Additional Information and Declarations

### Data Deposition

The following information was supplied regarding data availability: The raw data are provided in a Supplemental File.
