# Supplementary material for: Association of circulating microRNA-122 with presence and severity of atherosclerotic lesions
Source: PeerJ. 2018 Jul 4;6:e5218. doi: 10.7717/peerj.5218 (PMC6035718; doi:10.7717/peerj.5218)
Supplement: Supplemental Information 1 [file peerj-06-5218-s001.pptx]

## Slide 1
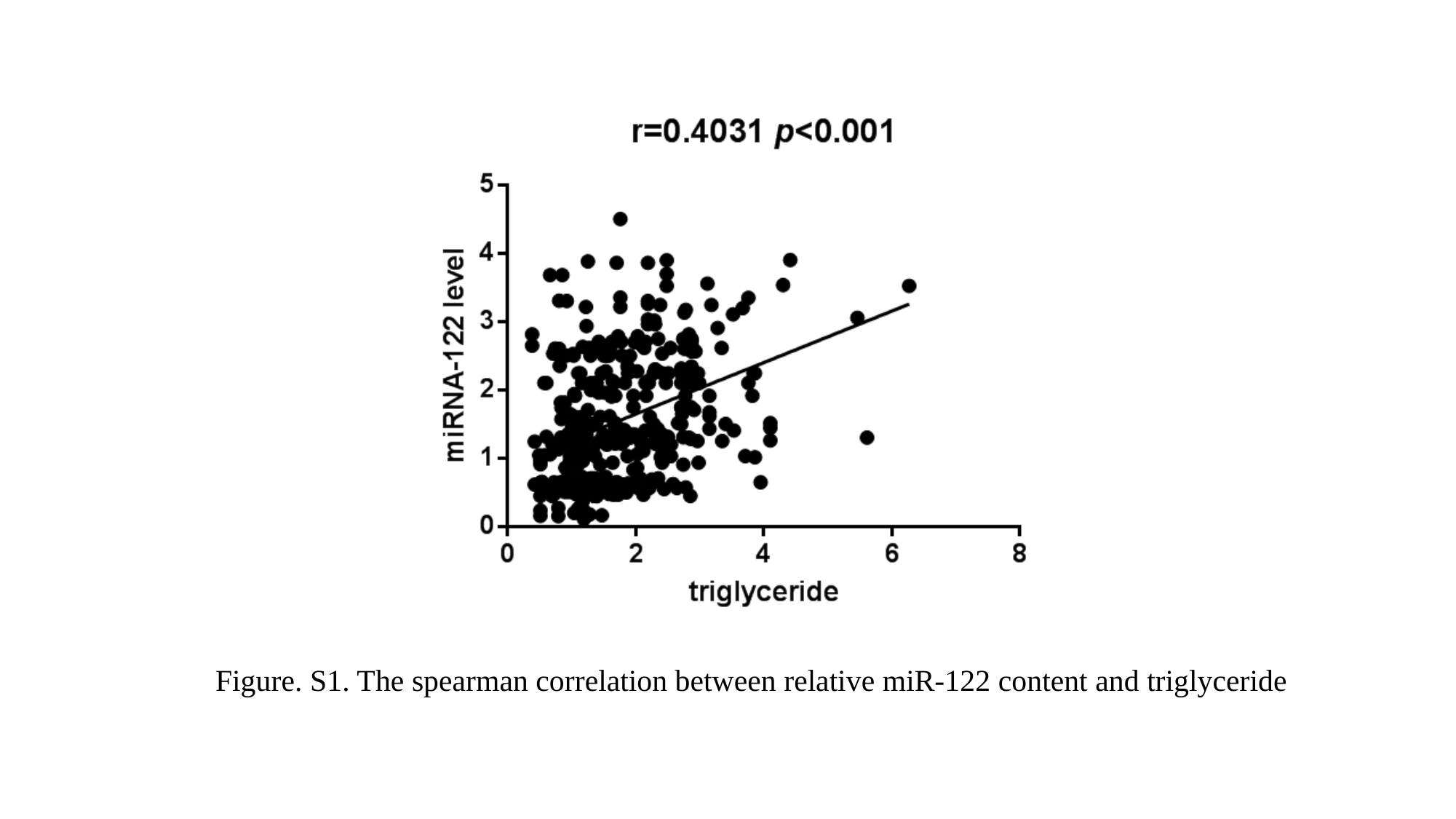

Figure. S1. The spearman correlation between relative miR-122 content and triglyceride

## Slide 2
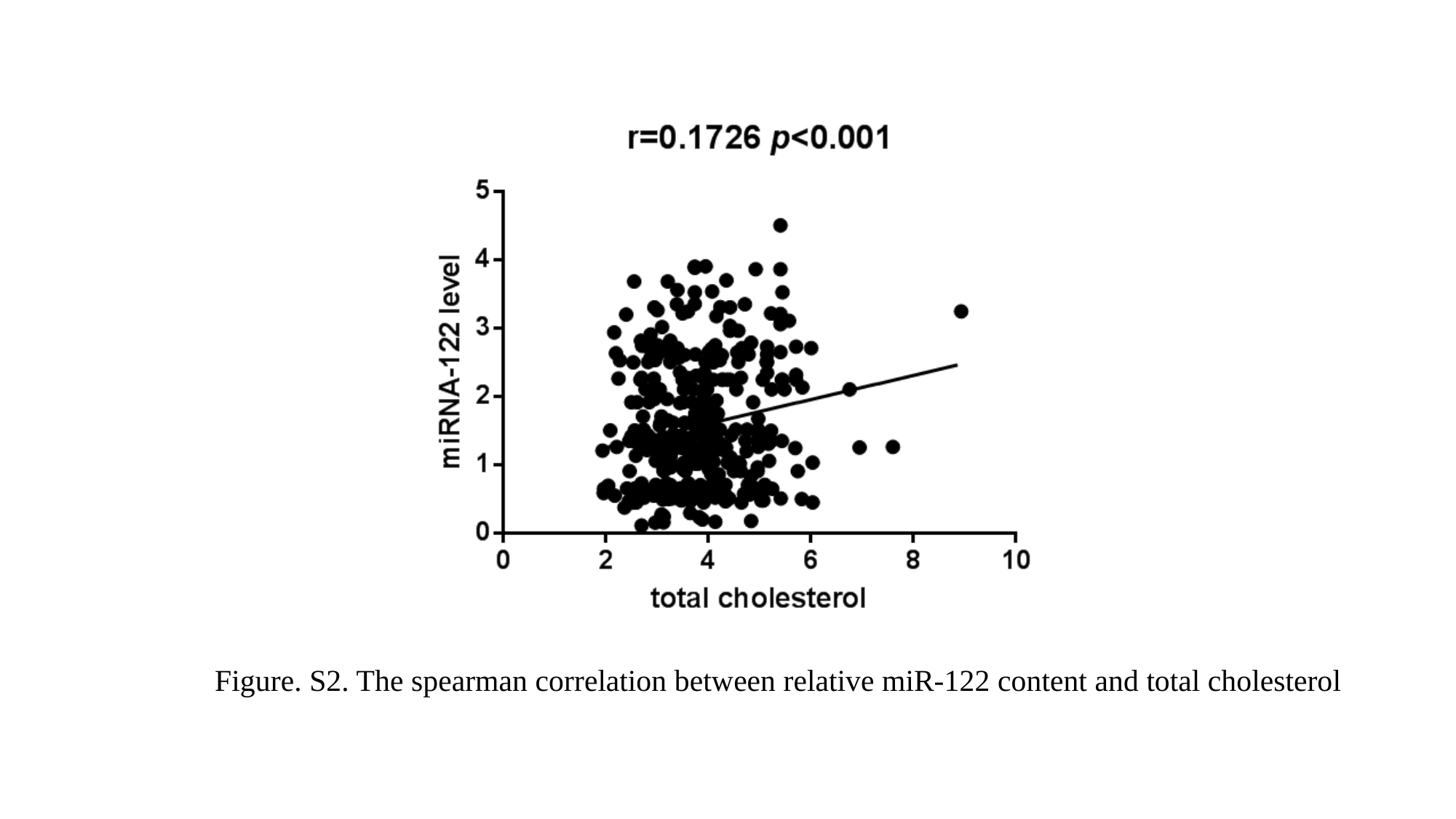

Figure. S2. The spearman correlation between relative miR-122 content and total cholesterol
